# Cancer detection via primary care urgent referral and association with practice characteristics: a retrospective cross-sectional study in England from 2009/2010 to 2018/2019

**DOI:** 10.3399/BJGP.2020.1030

**Published:** 2021-09-21

**Authors:** Thomas Round, Mark Ashworth, Veline L’Esperance, Henrik Møller

**Affiliations:** School of Population Health and Environmental Sciences, King’s College London; National Cancer Registration and Analysis Service, Public Health England, London.; School of Population Health and Environmental Sciences, King’s College London, London.; School of Population Health and Environmental Sciences, King’s College London, London.; School of Cancer and Pharmaceutical Sciences, King’s College London, London.

**Keywords:** cancer, early diagnosis, primary care, referral and consultation

## Abstract

**Background:**

There is substantial variation in the use of urgent suspected cancer referral (2-week wait [2WW]) between practices.

**Aim:**

To examine the change in use of 2WW referrals in England over 10 years (2009/2010 to 2018/2019) and the practice and population factors associated with cancer detection.

**Design and setting:**

Retrospective cross-sectional study of English general practices and their 2WW referral and Cancer Waiting Times database detection data (all cancers other than non-melanoma skin cancers) from 2009/2010 to 2018/2019.

**Method:**

A retrospective study conducted using descriptive statistics of changes over 10 years in 2WW referral data. Yearly linear regression models were used to determine the association between cancer detection rates and quintiles of practice and population characteristics. Predicted cancer detection rates were calculated, as well as the difference between lowest to highest quintiles.

**Results:**

Over the 10 years studied there were 14.89 million 2WW referrals (2.24 million in 2018/2019), and 2.68 million new cancer diagnoses, of which 1.26 million were detected following 2WW. The detection rate increased from 41% to 52% over the time period. In 2018/2019 an additional 66 172 cancers were detected via 2WW compared with 2009/2010. Higher cancer detection via 2WW referrals was associated with larger practices and those with younger GPs. From 2016/2017 onwards more deprived practice populations were associated with decreased cancer detection.

**Conclusion:**

From 2009/2010 to 2018/2019 2WW referrals increased on average by 10% year on year. The most consistent association with higher cancer detection was found for larger practices and those with younger GPs, though these differences became attenuated over time. The more recent association between increased practice deprivation and lower cancer detection is a cause for concern. The COVID-19 pandemic has led to significant impacts on 2WW referral activity and the impact on patient outcomes will need to be studied.

## INTRODUCTION

Most people with cancer present symptomatically to primary care,^1^ although the diagnosis of cancer in general practice is not straightforward.^2^^,^^3^ International variations in cancer survival have been partly attributed to healthcare system differences in primary care,^4^ particularly whether systems with prominent primary care ‘gatekeeping’ may result in longer diagnostic intervals and poorer outcomes for patients with cancer.^5^^,^^6^

Concerns about diagnostic delays led to the implementation of urgent referral pathways in England.^7^ These are based on National Institute for Health and Care Excellence (NICE) referral guidelines initially published in 2005,^8^ and updated in 2015.^9^ A primary care referral for suspected cancer aims for cases to be seen by a specialist or have a diagnostic test within 2 weeks of referral (2-week wait [2WW]). For many cancers there is good evidence that the time to diagnosis and treatment is reduced for patients who are referred urgently.^10^^,^^11^ There are significant variations in the use of 2WW between practices,^12^ with referral route an important potential predictor of time to diagnosis.^13^^,^^14^

Higher practice use of 2WW is associated with reduced cancer patient mortality,^15^ and in reduced late-stage cancers at diagnosis.^16^ A previous study found that higher practice referral rates for upper gastrointestinal endoscopy were also associated with improved patient outcomes.^17^ A cross-sectional study in 2013 of practice characteristics associated with use of both 2WW and endoscopy referrals suggested that practice-level attributes explained a substantial amount of between-practice variation.^18^

A more detailed analysis has been called for to understand the variation in the use of 2WW pathways,^7^^,^^12^^,^^19^ and the characteristics of primary care associated with higher practice cancer detection,^15^^,^^16^ including confirming whether associations found previously (for 2013)^18^ are consistent over longer and more recent time periods.

## METHOD

### Design and setting

This was a retrospective cross-sectional study of national cancer and general practice data in England for the financial years 2009/2010 to 2018/2019.

### National and practice cancer data

The national Cancer Waiting Times (CWT) system in England is used to monitor cancer waiting times targets, and is a record of cancer registration.^20^ This includes all patients diagnosed with cancer (International Classification of Diseases, 10th Revision [ICD-10] codes C00–C97), excluding non-melanoma skin cancer (C44). National-level data on cancer registrations and 2WW referrals are available from NHS England,^21^ while practice 2WW cancer detection rate data are available from Public Health England (PHE) National Cancer Registration and Analysis Service (NCRAS) ‘fingertips’ practice profiles,^22^ which are generated where the practice list size is at least 1000. Two-week wait referral metrics are available at a national and individual practice level and include detection rate — the proportion of CWT-recorded cancers (0% to 100%) resulting from a 2WW referral (that is, the sensitivity of referral); and conversion rate — the proportion of urgent referrals for suspected cancer that result in a diagnosis of cancer (that is, the positive predictive value for cancer among the patients referred).

**Table:** How this fits in

There is considerable variation in the use of urgent suspected cancer referrals (2-week wait [2WW]) between general practices in England, with increased use associated with improved outcomes for patients with cancer. There has been limited research into the practice and population characteristics associated with cancer detection via 2WW referral pathways. Over the 10-year period up to 2018/2019, yearly 2WW referrals more than doubled to more than 2.24 million, leading to an increase in cancer detection and 66 172 additional cancers diagnosed via 2WW in 2018/2019 compared with 2009/2010. Higher cancer detection via 2WW referrals was associated with larger practices and those with younger GPs, although the relationship with GP age was attenuated in more recent years. Of concern are decreases in 2WW referrals during the COVID-19 pandemic and the appearance of potential disparity in cancer detection, with lower rates in practices that serve more deprived populations.

This study focused on the CWT 2WW referral indicator of ‘cancer detection’ at a practice level (that is, the sensitivity of selection of patients for urgent referral), which is significantly associated with cancer patient outcomes.^16^

### Practice characteristics

Descriptive data for all practices in England were obtained from NHS Digital (https://digital.nhs.uk) for the 10 financial years studied, as described in previous publications.^23^^–^25 Data included workforce information (such as mean age of practice GPs), practice characteristics (such as list size), list size per full-time equivalent (FTE) GP, and the demographic characteristics of registered patients.

Estimates of the proportions of patients from ethnic minority groups were obtained for the location of each practice, adjusted for Lower Layer Super Output Area data for the practice postcode. Deprivation data for each general practice were attributed as the mean of the Index of Multiple Deprivation (IMD) scores weighted by the proportion of practice patients resident in each Lower Layer Super Output Area. Quality and Outcomes Framework (QOF) data were also obtained from NHS Digital, based on achievement of a series of targets relating to long-term condition management and public health goals.^26^ This study examined whether and how these practice and population characteristics were associated with cancer detection via 2WW referral pathways.

For 2018/2019, there were 6873 practices in England, with 92 (1.3%) excluded, mainly due to missing referral data, and 6781 practices included for analysis (see Supplementary Figure S1 for details). See Supplementary Table S1 for a detailed breakdown of exclusions from 2009/2010 to 2018/2019.

### Statistical analysis

Data on yearly cancer registrations and 2WW referral metrics were extracted from NHS England and PHE ‘fingertips’ for descriptive analysis. Variable descriptions and analysis were performed using Stata (version 16). Practice and population variables were stratified into quintiles (five equal groups) for analysis, similarly to previously described methods.^16^^,^^27^ Multicollinearity was tested using the variance inflation factor,^28^ which was <2 (mean 1.33) for all included variables, suggesting no significant multicollinearity. Linear and multiple linear regression models were used to explore the association between practice variable quintiles and cancer detection for each study year, starting from 2018/2019 and then the previous nine financial years. *P*-values were calculated for significance of differences in predicted cancer detection between variable quintiles. Values were expressed as percentage point differences between lowest (Q1) and highest (Q5) quintiles for each variable.

## RESULTS

### Cancer diagnoses and 2WW referral data 2009/2010 to 2018/2019

**Figure 1.**
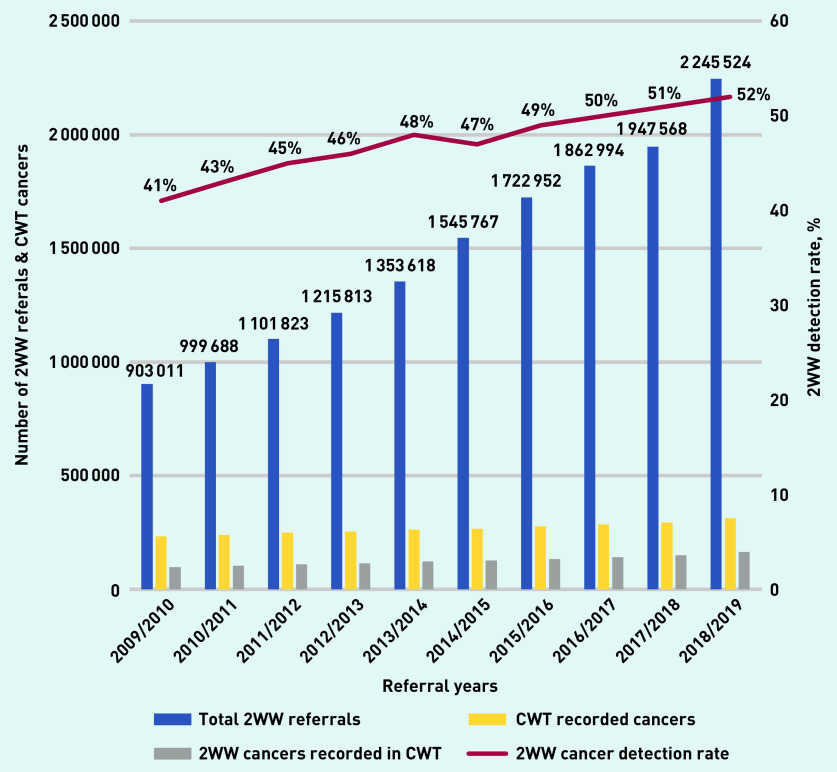
*Two-week wait referrals and detection rate (CWT database) in England 2009/2010 to 2018/2019. 2WW = 2-week wait. CWT = Cancer Waiting Times.*

Over the 10 years studied, there were 14.89 million 2WW referrals, and 2.68 million new cancer registrations, of which 1.26 million were detected via urgent referral (Table 1). Total 2WW referrals more than doubled to >2.24 million in 2018/2019 (Figure 1), with an average yearly increase of 10%. However, the yearly conversion rates decreased from 10.8% to 7.3% (see Supplementary Figure S2 for details). CWT database-registered cancers increased from 234 138 in 2009/2010 to 313 525 in 2018/2019, a 34% relative increase over 10 years (Table 1). Cancer detection via 2WW referral increased from 41% in 2009/2010 to 52% in 2018/2019, leading to an increase in cancers diagnosed following 2WW referral from 97 760 (2009/2010) to 163 932 (2018/2019). There were an additional 66 172 cancers detected via 2WW in 2018/2019 compared with 2009/2010 (a 68% relative increase). If cancer detection rate had been maintained at 41% (rather than yearly increases) 165 899 fewer cancers would have been detected via 2WW over the 10-year period.

**Table 1.** Practice cancer referral data 2009/2010 to 2018/2019

**Financial year**	**Total 2WW referrals, *n* (% yearly change)**	**CWT recorded cancers, *n* (% yearly change)**	**2WW cancers recorded in CWT, *n* (% yearly change)**	**2WW cancer detection rate, % (% yearly change)**	**2WW cancer conversion rate, %**
2009/2010	903 011	234 138	97 760	41	10.8
2010/2011	999 688 (11)	240 572 (3)	103 023 (5)	43 (5)	10.3
2011/2012	1 101 823 (10)	250 456 (4)	110 400 (7)	45 (5)	10.0
2012/2013	1 215 813 (10)	254 061 (1)	114 945 (4)	46 (2)	9.5
2013/2014	1 353 618 (11)	262 414 (3)	122 229 (6)	48 (4)	9.0
2014/2015	1 545 767 (14)	266 723 (2)	126 637 (4)	47 (−2)	8.2
2015/2016	1 722 952 (11)	276 555 (4)	133 958 (6)	49 (4)	7.8
2016/2017	1 862 994 (8)	284 655 (3)	141 790 (6)	50 (2)	7.6
2017/2018	1 947 568 (5)	294 514 (3)	149 046 (5)	51 (2)	7.7
2018/2019	2 245 524 (15)	313 525 (6)	163 932 (10)	52 (2)	7.3
10-year total number of referrals or cancers	14 898 758	2 677 613	1 263 720	—	—
Change in financial year 2018/2019 compared with 2009/2010	1 342 513 149% relative (increase)	79 387 additional cancers (34% relative increase)	66 172 additional cancers (68% relative increase)	11% increase (27% relative increase)	3.5% decrease (48% relative decrease)

*2WW = 2-week wait. CWT = Cancer Waiting Times database.*

### Variable summary

The distribution of practice and population characteristics for the practices included are summarised in Table 2. Over the 10 years studied there was a reduction in the number of practices, with consequent increase in list size (from 6910 to 8717). There was an increase in the average registered patients per FTE GP to >2300, although the method of recording this variable changed in 2015/2016.^29^ GP mean age (range 47.0–47.9 years), IMD score (range 25.6–26.2), and the proportion of registered patients aged ≥65 years (range 16%–17%) were relatively stable over this time period, while the proportion of registered patients of white ethnicity decreased from 89% to 83% over the study period. Maximum available practice QOF points decreased over time from 1000 (2009/2010) to 559 (2014/2015 onwards).

**Table 2.** GP practice characteristics in England 2009/2010 to 2018/2019 included in analysis

	**2009/2010**	**2010/2011**	**2011/2012**	**2012/2013**	**2013/2014**	**2014/2015**	**2015/2016**	**2016/2017**	**2017/2018**	**2018/2019**
**Practices, *n***	7717	7730	7725	7739	7706	7428	7446	7233	6987	6781
**2WW detection rate, %**	41	43	45	46	48	47	49	50	51	52
**Practice list size, mean, *n***	6910	6973	7050	7145	7230	7491	7669	7690	8307	8717
**List per FTE GP, mean, *n***	1808	1851	1824	1847	1851	1879	2339	2342	2358	2304
**GP average age, years, mean**	47.6	47.5	47.4	47.8	47.9	47.6	47.1	47.3	47.7	47.0
**QOF score, mean (maximum points available)**	958 (1000)	950 (1000)	973 (1000)	964 (1000)	844 (900)	531 (559)	535 (559)	540 (559)	539 (559)	540 (559)
**Practice IMD score, mean**	25.9	26.0	25.8	26.2	26.2	26.1	26.1	26.1	26.1	25.6
**Patients aged >65 years, %**	16	16	16	17	16	16	16	16	17	17
**Ethnicity, white, %**	89	88	83	83	84	85	84	83	84	83

*2WW = 2-week wait. FTE = full-time equivalent. IMD = Index of Multiple Deprivation. QOF = Quality and Outcomes Framework.*

### Cancer detection and predictors 2018/2019

The univariable linear regression associations between practice characteristic quintiles and cancer detection are shown in Table 3. Variables positively associated with higher cancer detection rates included practice list size (+4 percentage points higher cancer detection from Q1 to Q5), QOF score (+2), proportion of patients aged >65 years (+5), and proportion of white patients (+3). As an example, cancer detection rates increased from 50% for practices in the lowest quintile of list size (mean 3207 patients) to 54% for practices in the highest quintile (mean 16 847 patients). Variables negatively associated with cancer detection rates included registered patients per FTE GP (−2 percentage points difference from Q1 to Q5), GP average age (−3), and IMD score (−5).

**Table 3.** Predicted cancer detection for variable quintiles 2018/2019 for both linear and multiple linear regression, and percentage point difference in predicted cancer detection from Q1 to Q5

**Practice variable quintiles**	**2018/2019 practice variable quintile, mean**	**Linear regression**		**Multiple linear regression**	
		**Predicted cancer detection, % (95% CI)**	**Q1–Q5 percentage point difference**	**Predicted cancer detection, % (95% CI)**	**Q1–Q5 percentage point difference**
**Practice list size (mean 8717)**					
1	3207	50 (49 to 50)	—	51 (50 to 51)	—
2	5423	52 (51 to 52)	—	52 (52 to 53)	—
3	7686	53 (53 to 54)	—	53 (52 to 54)	—
4	10 427	53 (53 to 54)	—	53 (42 to 43)	—
5	16 847	54 (53 to 54)	—	53 (42 to 43)	—
	*P*<0.001	—	+4	*P*<0.001	+2

**List per FTE GP (mean 2304)**					
1	1297	53 (53 to 54)	—	53 (52 to 54)	—
2	1566	54 (53 to 55)	—	53 (53 to 54)	—
3	1934	52 (52 to 53)	—	52 (52 to 53)	—
4	2403	51 (50 to 52)	—	51 (51 to 52)	—
5	4468	51 (51 to 52)	—	52 (52 to 53)	—
	*P*<0.001	—	−2	*P* = 0.002	−1

**GP average age, years (mean 47)**					
1	38.9	53 (52 to 54)	—	53 (52 to 54)	—
2	42.9	54 (53 to 54)	—	53 (52 to 54)	—
3	46.2	53 (52 to 54)	—	53 (52 to 53)	—
4	50.3	52 (51 to 53)	—	52 (51 to 53)	—
5	54.2	50 (49 to 51)	—	51 (51 to 52)	—
	*P*<0.001	—	−3	*P* = 0.002	−2

**QOF score, mean (mean 540.1)**					
1	495.5	51 (50 to 51)	—	52 (51 to 53)	—
2	540.4	52 (51 to 53)	—	53 (52 to 53)	—
3	550.2	52 (52 to 53)	—	52 (51 to 53)	—
4	554.1	53 (53 to 54)	—	53 (52 to 53)	—
5	558.4	53 (53 to 54)	—	53 (53 to 53)	—
	*P*<0.001	—	+2	*P*= 0.113	+1

**IMD score 2019 (mean 25.6)**					
1	6.9	54 (54 to 55)	—	54 (53 to 54)	—
2	14.1	54 (53 to 55)	—	54 (53 to 54)	—
3	21.8	53 (52 to 53)	—	53 (52 to 53)	—
4	32.1	51 (51 to 52)	—	52 (51 to 52)	—
5	53.2	49 (49 to 50)	—	50 (50 to 51)	—
	*P*<0.001	—	−5	*P*<0.001	−4

**Patients aged >65 years (mean 17%)**					
1	8%	50 (50 to 51)	—	51 (50 to 52)	—
2	13%	51 (51 to 52)	—	52 (51 to 53)	—
3	17%	52 (52 to 53)	—	52 (52 to 53)	—
4	20%	53 (52 to 53)	—	52 (52 to 53)	—
5	26%	55 (54 to 56)	—	54 (54 to 55)	—
	*P*<0.001	—	+5	*P*<0.001	+3

**Ethnicity, white (mean 83%)**					
1	49%	51 (50 to 51)	—	53 (52 to 54)	—
2	80%	52 (51 to 53)	—	53 (52 to 53)	—
3	92%	52 (52 to 53)	—	52 (52 to 53)	—
4	97%	53 (52 to 54)	—	52 (51 to 53)	—
5	98%	54 (53 to 54)	—	52 (51 to 53)	—
	*P*<0.001	—	+3	*P*= 0.092	−1

*CI = confidence interval. FTE = full-time equivalent. IMD = Index of Multiple Deprivation. QOF = Quality and Outcomes Framework.*

For the multivariable linear regression models, all variables remained significantly associated (consistently positively or negatively) with cancer detection rates, with the exception of QOF score and patient ethnicity (Table 3). Overall, differences in detection rate between Q1 and Q5 were attenuated after adjustment for covariates. The largest cancer detection rate Q1 to Q5 differences were found for practice list size (+2), GP average age (−2), deprivation (−4), and older patients (+3).

### Difference in predicted cancer detection rates between practice variable quintiles 2009/2010 to 2018/2019

Cancer detection analyses for quintiles of practice characteristics in univariable and multiple linear regression models were carried out for each previous financial year from 2018/2019 to 2009/2010 (yearly data tables are available from the authors on request). The percentage point difference in cancer detection rates for practice variables between Q1 and Q5 for each year is described in Tables 4 and 5, obtained from univariable and multiple linear regression models, respectively.

**Table 4.** Percentage difference in predicted cancer detection from Q1 to Q5 in linear regression models, years 2009/2010 to 2018/2019 (all statistically significant, *P*<0.001)

	**2009/2010**	**2010/2011**	**2011/2012**	**2012/2013**	**2013/2014**	**2014/2015**	**2015/2016**	**2016/2017**	**2017/2018**	**2018/2019**
**Practice list size**	+5	+6	+4	+3	+5	+4	+5	+4	+4	+4
**List per FTE GP**	−6	−4	−4	−2	−3	−3	−2	−2	−1	−2
**GP average age, years**	−7	−7	−6	−6	−5	−5	−4	−4	−3	−3
**QOF score**	+5	+4	+3	+2	+2	+3	+2	+2	+2	+2
**IMD score**	−3	−3	−2	−1	−3	−3	−3	−4	−5	−5
**Patients aged >65 years**	+5	+5	+2	+2	+3	+3	+3	+3	+3	+5
**Ethnicity, white**	+6	+5	+2	+2	+3	+3	+2	−1	+2	+3

*FTE = full-time equivalent. IMD = Index of Multiple Deprivation. QOF = Quality and Outcomes Framework.*

**Table 5.** Percentage point difference in predicted cancer detection from Q1 to Q5 in multiple linear regression models, years 2009/2010 to 2018/2019^a^

	**2009/2010**	**2010/2011**	**2011/2012**	**2012/2013**	**2013/2014**	**2014/2015**	**2015/2016**	**2016/2017**	**2017/2018**	**2018/2019**
**Practice list size**	+2	+2	+2	+1	+3	+3	+3	+4	+2	+2
**List per FTE GP**	−3	0^b^	−2	−1	−1	−1^b^	−1^b^	−1^b^	−1^b^	−1
**GP average age, years**	−6	−6	−4	−5	−3	−4	−3	−3	−2	−2
**QOF score**	+3	+3	+1	0	+1	+1	+1	+1^b^	+1	+1^b^
**IMD score**	−1^b^	−1^b^	0^b^	−1^b^	−2^b^	−1^b^	−1^b^	−4	−4	−4
**Patients aged >65 years**	+2	+2	−1	+1	−1^b^	+2	+2	+2	+2	+3
**Ethnicity, white**	+2	+3	+1	−1^b^	+2	+1	−1	−2	−1	−1^b^

a *All statistically significant at* P*<0.001 unless otherwise stated.*

b *Not statistically significant. FTE = full-time equivalent. IMD = Index of Multiple Deprivation. QOF = Quality and Outcomes Framework.*

In the linear regression models (Table 4) increasing numbers of patients per FTE GP, GP average age, and practice deprivation (IMD score) all had significant negative associations with cancer detection. Although the Q1 to Q5 difference from 2009/2010 to 2018/2019 reduced for patients per FTE GP (from −6 to −2) and GP average age (−7 to −3), for deprivation the quintile difference increased (from −3 to −5). Positive associations with increased cancer detection rates were found for increased practice list size, QOF score, and the proportion of patients aged >65 years.

In the multiple linear regression models (Table 5), the most consistent association with increased cancer detection was for larger practices, with younger GPs serving older populations. Over the study period differences in cancer detection between Q1 and Q5 for GP age became less pronounced (from −6 in 2009/2010 to −2 in 2018/2019), while deprivation initially lacked significance, but was significantly associated with reduced cancer detection (−4 percentage point difference) from 2016/2017 onwards.

### Excluded practices

See Supplementary Table S2 for the characteristics of excluded practices, including those with missing referral data. The total number of excluded practices reduced from 588 (2009/2010) to 92 (2018/2019). They were smaller (mean list size <3000), had older GPs (mean age 52 years), lower QOF scores, and served younger and more deprived, diverse populations.

## DISCUSSION

### Summary

Two-week wait referrals in England more than doubled over the 10-year time period studied to >2.24 million in 2018/2019, with a subsequent increase in cancer detection rates (41% to 52%), and decrease in conversion rate (10.8% to 7.3%). This led to 66 172 additional cancers detected following 2WW referral in 2018/2019 compared with 2009/2010.

Predicted 2WW cancer detection was consistently associated with larger practice list size (positive association) and increasing GP age (negative association), although over the study period there was a narrowing in the difference of cancer detection between practices with younger and older GPs. In the last 3 years of study, cancer detection rates were consistently lower in more deprived areas, an effect not seen in earlier years.

### Strengths and limitations

The strengths of this study are using large national databases in England including all 2WW referrals and cancers registered in the CWT database, and data on most practices in England over 10 years.

The CWT database contains diagnosis and treatment information on patients with cancer who were offered treatment in the NHS, whichever diagnosis route they came through.^20^^,^^21^ However, not all patients with cancer are included in the CWT. Data from adults diagnosed with colorectal, lung, or ovarian cancer in England (2009–2013) were linked in a study from CWT to cancer registry, mortality, and Hospital Episode Statistics data, which found that approximately 80% of patients were included.^30^ Patients not recorded in the CWT are more likely to be in the youngest or oldest age groups, have increased comorbidity, and be diagnosed through emergency presentation routes. They are also more likely to have late or missing stage cancer, and have in general poorer survival. NCRAS routes to diagnosis data include cancer registrations following GP referral (2WW or routine), screening, outpatient or inpatient elective, death certification, and diagnosed within 28 days of emergency activity.^31^ The most recent data to 2017^32^ showed that more than half of all cancers are diagnosed after a GP referral, and there have been reductions in diagnosis after emergency activity to below 20% of all cancers. Approximately 4% of cancer registrations are via an unknown route.

Practice-level characteristics were extracted from quality-assured national published datasets,^26^^,^^33^ using established methodology.^24^^,^^25^ The authors had no access to patient-level data, so could not adjust for the characteristics of patients who were investigated or referred at the individual level, although practice-level measures of ethnicity and deprivation were used and adjusted for Lower Layer Super Output Area of residence of the registered population. The studied associations may be affected by practices not included in the analyses, although this reduced over time to <2% (see Supplementary Table S2 for details). The number of practices with missing 2WW referral data also reduced over the 10 years studied from 3.2% to 0.5%, suggesting increased robustness of the PHE NCRAS data. There were also changes over the time period studied in how some practice characteristics were calculated, such as in 2015/2016 NHS England changed how FTE GP was calculated.^29^

Variation in practice with respect to cancer referrals is more complex than publicly reported metrics suggest (including detection rate),^34^^,^^35^ and no single indicator captures quality of care.

### Comparison with existing literature

There have been substantial increases in the proportion of patients with cancer diagnosed following 2WW referral and a subsequent decrease in those diagnosed via emergency routes,^36^ in whom there are worse outcomes.^31^ There are also large differences in the number of 2WW referrals based on cancer site and patient demographics.^37^ Following revised NICE 2WW referral guidance in 2015,^38^ there have been further substantial increases in referrals and related pressure on diagnostic services and diagnostic intervals.^39^

Previous studies have used data from a single year (2013) and examined the association between practice 2WW and endoscopy use with GP patient survey data^40^ and general practice characteristics.^18^ Practice-level attributes explained a substantial amount of between-practice variance in 2WW but little of the variance in endoscopy, with urgent referral found to be higher in training practices^41^ and those with younger GPs.^18^

There have been several studies into 2WW referral metrics, including detection rate, which have found year-on-year random variation,^19^ with significant observed differences in case-mix,^42^ and variation in referral selection accuracy and thresholds.^43^ GPs and practices are not working in isolation, and there are influences from the wider healthcare system.^34^^,^^35^ Research has shown that GPs in England are potentially less likely to investigate and refer for suspected cancer than GPs in similar high-income countries.^6^ There is also an association between practice 2WW referral metrics and individual GP referral thresholds.^44^

The broader literature on referrals suggests that much variation is unexplained,^45^^,^^46^ with conflicting evidence about the relationship between practice size and GP characteristics on referral rates,^45^^,^^47^ and the impact of GP and practice characteristics on potential delays in cancer diagnosis.^48^ The current study suggests that GP age has become less important as a predictor of cancer detection over time. One explanation is the diffusion of NICE referral guidance into clinical practice of individual GPs,^10^ with GPs of all ages more likely to follow guideline-compliant practice.

A previous study found that older patients (aged >85 years) and more deprived patients were less likely to be referred,^49^ although there has been conflicting evidence at a practice level.^45^^,^^50^ The current study’s findings of an association between practice deprivation and reduced cancer detection in recent time periods is a concern, particularly given continuing evidence for the persistence of the ‘inverse care law’.^51^^,^^52^

### Implications for research and practice

This study has shown a substantial increase in 2WW referrals to >2.24 million per year in 2018/2019. The average English general practice makes >300 2WW referrals per year or approximately 65 per FTE GP. This has been associated with an increase in the proportion of cancers detected following GP 2WW referral from 41% to 52%, and a reduction in the 2WW conversion rate from more than 10% to 7%. This increase in referral activity has likely led to improved cancer patient outcomes.^16^ Health service waiting time targets are considered to be important indicators of the quality of cancer care,^45^ and, even after a period of constrained resources and increases in referrals, the English healthcare system maintained (in 2018/2019) 92% of urgently referred patients being seen by a specialist within 2 weeks of referral.^21^

For the more recent financial year (2019/2020)^53^ there were 2 386 815 2WW referrals in England with overall detection rate further increasing to 53.5% and conversion rate to 6.7%,^53^ with the largest numbers of referrals for suspected skin (*n* = 509 668), lower gastrointestinal (*n* = 443 534), and breast (*n* = 435 253) cancers. PHE NCRAS has published 2WW pathway specific detection and conversion rates,^54^ with substantial variation between different 2WW pathways. This suggests that some pathways are working more effectively than others, with international evidence showing the UK cancer survival gap (between other comparable countries) reducing for breast cancer but not for some other cancers.^4^ This suggests scope for focusing on specific cancer types where increased triage testing and diagnostic access in primary care might make the most impact.^16^

The most recent (2019/2020) conversion rate for all 2WW referrals in England was 6.7%,^53^ in other words, roughly 7 in 100 referrals are diagnosed with cancer. While the NICE 2WW guidelines in 2015^9^ specified a risk threshold to consider referral of 3%, patients would opt for investigation or referral at a lower risk threshold of 1%.^55^ With longer-term aims of faster diagnostic standards and a greater proportion of early-stage diagnoses,^56^ there are clear issues around finite staff, diagnostic access, and capacity across primary and secondary care.^57^^–^59 This includes further research to understand the health economic impacts of increasing referrals and reduced referral thresholds, and the potential risks of this including patient anxiety, iatrogenic harms, and overdiagnosis.

During the COVID-19 pandemic there have been reductions in cancer screening, 2WW referrals, and access to diagnostic tests, with likely negative impacts on cancer diagnoses and outcomes.^60^^,^^61^ It is therefore imperative to maintain gains made over the last 10 years in the use of suspected cancer pathways. This includes clear and consistent messaging to the public that they should contact primary care services if they have worrying symptoms, and that, while primary care is under pressure, assessment and urgent referral pathways are continuing, although care may be delivered in a different way (for example, telephone and online consultations).^62^^,^^63^

Over the 10-year study period there has been a consistent significant association between lower cancer detection and smaller practices with older GPs, although over time there was less observed variation, particularly with GP age. The more recent significant association between increased practice deprivation and lower cancer detection is a cause for concern. Further research to better understand the significance of these findings for primary care staff and their patients is called for, including potential interventions to continue to facilitate cancer detection via primary care referral, particularly given the recent impacts of the COVID-19 pandemic.

## Data

The analysis is based on routine practicelevel data from the NHS. Practice-level referral data is available from PHE fingertips.^22^
